# The Intracellular Transporter AtNRAMP6 Is Involved in Fe Homeostasis in *Arabidopsis*

**DOI:** 10.3389/fpls.2019.01124

**Published:** 2019-09-17

**Authors:** Jiyu Li, Yuerong Wang, Lu Zheng, Yun Li, Xueli Zhou, Jingjun Li, Dongfang Gu, Ending Xu, Yaping Lu, Xi Chen, Wei Zhang

**Affiliations:** ^1^Department of Biochemistry & Molecular Biology, College of Life Sciences, Nanjing Agricultural University, Nanjing, China; ^2^Institute of Horticulture, Anhui Academy of Agricultural Sciences, Hefei, China; ^3^Key Laboratory of Genetic Improvement and Ecophysiology of Horticultural Crops, Hefei, China

**Keywords:** *Arabidopsis thaliana*, AtNRAMP6, Golgi, *trans*-Golgi network, lateral root growth, Fe homeostasis

## Abstract

Natural resistance-associated macrophage proteins (NRAMPs) have been shown to transport a wide range of divalent metal ions, such as manganese (Mn), cadmium (Cd), and Iron (Fe). Iron is an essential micronutrient for plants and Fe deficiency can lead to chlorosis or decreased biomass. AtNRAMP6 has demonstrated the capability to transport Cd, but its physiological function is currently unclear. This study demonstrates that AtNRAMP6 is localized to the Golgi/*trans*-Golgi network and plays an important role in intracellular Fe homeostasis in the flowering plant genus *Arabidopsis*. GUS tissue-specific expression revealed that *AtNRAMP6* is highly expressed in the lateral roots and young leaves (three to four top leaves) of *Arabidopsis*. Moreover, knocking out *AtNRAMP6* was shown to impair lateral root growth without having a differential effect on the main root under Fe-deficient conditions. Lastly, the expression of *AtNRAMP6* was found to exacerbate the sensitivity of the yeast mutant *Δccc1* to an excessive amount of Fe. These findings indicate that AtNRAMP6 plays an important role in the growth of *Arabidopsis* in Fe-deficient conditions.

## Introduction

Iron (Fe) is a vital micronutrient for the survival of all organisms. In plants, Fe is used in cellular respiration, photosynthetic electron transport, biosynthesis of chlorophyll, and various other metabolic functions (Bashir et al., 2016). Although Fe is abundant in the earth’s crust, Fe availability often limits plant growth due to the low solubility of Fe in aerobic environments (Grotz and Guerinot, 2006). The most obvious consequence of Fe deficiency in plants is chlorosis due to a decrease in chlorophyll content, which significantly affects plant growth, development, and product quality (Bashir et al., 2016). In order to deal with Fe deficiency, plants have evolved a dual mechanism for obtaining Fe from the soil, whereby ferric Fe(III) is taken up as complex with organic compounds in graminaceous species, and ferrous iron (Fe^2+^) is directly taken up by Fe(II) transporters in the other species (Grotz and Guerinot, 2006). Once Fe has been transported into the cytosol of the plant, it is sent to proteins and organelles for use or storage (Hell and Stephan, 2003).

Transporters play a crucial role in Fe uptake, in detoxification, and in the maintenance of Fe homeostasis. A variety of transporters that may transport Fe have been identified in plants, such as cation diffusion facilitators (CDFs), natural resistance-associated macrophage proteins (NRAMPs), ZRT-IRT-like proteins (ZIPs), and yellow stripe–like proteins (YSLs) (Williams et al., 2000; Hall and Williams, 2003). The Fe-regulated transporter IRT1 is a major high-affinity Fe uptake transporter in Fe-limited conditions (Vert et al., 2002; Barberon et al., 2011). In seeds, the tonoplast VIT1 transporter mediates the import of Fe into the vacuole (Kim et al., 2006). Two chloroplast envelope-localized transporters, AtYSL4 and AtYSL6, play a role in detoxifying Fe by controlling the release of Fe from the chloroplast (Divol et al., 2013).

NRAMPs constitute an evolutionarily conserved family that functions as proton-coupled metal ion transporters that can transport Mn^2+^, Fe^2+^, Zn^2+^, Cu^2+^, Cd^2+^, Al^3+^, Co^2+^, and Ni^2+^ in prokaryotic and eukaryotic organisms (Gunshin et al., 1997; Maser et al., 2001; Nevo and Nelson, 2006). NRAMPs have been characterized as playing a critical role in Fe transport in plants. Three genes in rice, *OsNRAMP1*, *OsNRAMP5*, and *OsNRAMP6*, have been confirmed to be involved in Fe transport. *OsNRAMP1* is a plasma membrane–localized transporter that participates in cellular Fe, Cd, and arsenic (As) transport within plants (Curie et al., 2000; Nevo and Nelson, 2006; Takahashi et al., 2011). *OsNRAMP5* encodes plasma membrane–localized proteins and plays an important role in Mn, Fe, and Cd uptake, along with translocation in rice (Ishimaru et al., 2012; Sasaki et al., 2012; Yang et al., 2014). *OsNRAMP6* was found to act as a Fe and Mn transporter and contributes to disease resistance in rice (Peris-Peris et al., 2017).

In *Arabidopsis*, six genes encode members of the NRAMP transporter family. *AtNRAMP1* is expressed in the plasma membrane of root cells and functions as a high-affinity Mn transporter for Mn uptake under Mn deficiency (Cailliatte et al., 2010). It also cooperates with IRT1 for Fe uptake in the roots under sufficient metal conditions (Castaings et al., 2016). AtNRAMP2 is a *trans*-Golgi network (TGN)–localized manganese transporter, and the mutation of *AtNRAMP2* reduces root growth and disturbs photosynthesis and cellular redox homeostasis under manganese deficiency (Alejandro et al., 2017; Gao et al., 2018). AtNRAMP3 and AtNRAMP4 are localized to the tonoplast and play crucial roles both in the release of Fe from vacuoles during seed germination under Fe deficiency and in the export of vacuolar Mn in photosynthetic tissues of adult plants under Mn deficiency (Thomine et al., 2003; Lanquar et al., 2005; Lanquar et al., 2010; Mary et al., 2015). *AtNRAMP5* has not been functionally characterized yet. All these findings indicate that NRAMPs have diverse functions in the transport of various metals.

A previous study has shown that AtNRAMP6 contributes to Cd toxicity and is localized to an endomembrane of yeast (Cailliatte et al., 2009). However, its specific localization in *Arabidopsis* and its potential role in the transport of other metal ions are unknown. This study demonstrates that AtNRAMP6 is a Golgi/TGN-localized transporter in *Arabidopsis* and is specifically expressed at the lateral root. *Arabidopsis nramp6* mutant and complementation lines were also generated, and their analyses revealed that AtNRAMP6 plays an important role in lateral root growth in Fe-deficient conditions.

## Materials and Methods

### Plant Materials and Growth Conditions

The *Arabidopsis* T-DNA insertion mutant *nramp6-1*(GABI-Kat line ID 550D06) was obtained from The *Arabidopsis* Information Resource (TAIR) for this study. Homozygous mutant plants were genotyped by PCR using the gene primers N6-F (5′-TCTCTTTGTTCCTCAGTTGA-3′), N6-R (5′-GAAAGTTAATCATCATTGCCTCG-3′), and the T-DNA specific primer RB (5′-GGTGGATTTATCACAAATGGGAC-3′). To generate the complemented *nramp6* lines, the *AtNRAMP6* open reading frame was amplified with the primers 5′-ACTAGTATGGCGGCTGAAACAGCAAGT-3′ and 5′-ACTAGTTCAATTTAAGTCTCCTATAACCGCTAC-3′ and then subcloned into the pCAMBIA1304 vector using translational fusion with the cauliflower mosaic virus (CaMV) 35S promoter, and then the constructs were introduced into the *nramp6* mutant using the EHA105 strain of *Agrobacterium tumefaciens* following the floral dip protocol (Clough, 2005). The expression of *AtNRAMP6* was estimated using RT-PCR.

The Hoagland solution (5mM CaNO_3_, 5mM KNO_3_, 2mM MgSO_4_, 1mM NH_4_H_2_PO_4_, 0.185mM H_3_BO_3_, 2μM [NH_4_]_6_Mo_7_O_24_, 36.6μM MnSO_4_, 3μM ZnSO_4_, 1.28μM CuSO_4_, 40μM Fe[III]-EDTA) was used for *Arabidopsis* plant growth in this study. For plant growth in agar plates, seeds were surface-sterilized and sown with a one-half-strength Hoagland nutrient solution (with different iron concentration) containing a medium with 2% sucrose and 1% agar that was buffered with 0.5 g·L^−1^ MES at pH 5.7. The plates were placed in the dark at 4°C for 2 days and then grown in a growth chamber under 16 h of light (300 μmol s^−1^m^−2^) at 22°C and 8 h of darkness at 20°C with 60% relative humidity. For experiments investigating the metal content in plants, seeds were first sown on agar plates for 2 weeks and then transferred to hydroponic cultivation (one-quarter-strength Hoagland nutrient solution). After 1 week, the plants were exposed to Fe-deficient conditions for 1 week, and the tissues were harvested and dried. Hydroponically grown plants were cultivated under 14 h of light (300 μmol s^−1^m^−2^) at 22°C and 10 h of darkness at 20°C with 60% relative humidity.

### Gene Expression

Total RNA was extracted from different plant organs by using a RNeasy Plant RNA Extraction Kit (TaKaRa). Then, 1 μg of total RNA was used to synthesize the first-strand cDNAs using the PrimeScript™ RT Reagent Kit (TaKaRa). Quantitative real-time PCR reactions were performed in a 96-well plate using a CFX 96 fluorescent quantitative PCR apparatus from Bio-Rad with SYBR Premix Ex Taq™ (TaKaRa). For the expression pattern of *AtNRAMP6*, different tissues of 3-week-old WT plants were excised for RNA extraction. For the expression analysis of the *AtNRAMP6* response to different levels of metal exposure, 3-week-old WT plants were grown in a one-quarter-strength Hoagland nutrient solution with different concentrations of Fe, Mn, and Zn for 7 days, and then, the roots and shoots were sampled for RNA extraction. The primers for the expression analysis were using the following gene-specific primers (specific to the full-length *AtNRAMP6* transcript): RT*NR6*-F: 5′-GTAATGCCTCAGATTTGAGTCCA-3′; RT*NR6*-R: 5′-GGTTATGGTTGAACTCTGACCG-3′; *Actin2* was used as an internal control. *Actin*-F: 5′-GGTAACATTGTGCTCAGTGGTGG-3′; *Actin*-R: 5′-AACGACCTTAATCTTCATGCTGC-3′.

### Subcellular Localization

The coding sequence without the stop codon of *AtNRAMP6* was amplified and inserted into the BamHI and KpnI sites of the pXZP008-GFP vector (Shi et al., 2012). The *AtNRAMP6-GFP* construct was co-expressed with the markers mRFP-SYP61 (trans-Golgi), mRFP-Man1 (cis-Golgi), ST-mCherry (Golgi), or mRFP-ARA7 (PVC) (Saint-Jore et al., 2002; Shen et al., 2013; Cui et al., 2014) into *Arabidopsis* mesophyll protoplasts for fluorescence observation using a confocal laser scanning microscopy (confocal system-UltraVIEW VoX, Perkin Elmer). The excitation and emission wavelengths used to observe green fluorescence are 488 nm and 507–510 nm, respectively. The excitation and emission wavelengths used to observe red fluorescence are 584 nm and 607–610 nm, respectively. Mesophyll protoplasts were isolated from the leaves of 4-week-old plants according to the method described previously (Yoo et al., 2007), and the protoplasts were transfected with 10 μg of plasmid DNA using the polyethylene glycol method.

### GUS Histochemical Analysis

A 1.8-kb promoter region located upstream from the translation initiation codon of *AtNRAMP6* was amplified and inserted into the pCAMBIA1305 vector to form the Pro*NRAMP6*:GUS construct. The construct was transformed into Col WT plants using the EHA105 strain of *Agrobacterium tumefaciens* following the floral dip protocol as described above. T3 homozygous transgenic plants were used to analyze the GUS activity. Different tissues were immersed into the GUS Assay Buffer (50 mM Na_3_PO_4_, 1 mM ferrocyanide, 1 mM ferricyanide, and 0.2% Triton X-100, pH 7.2) containing 2 mM X-Gluc, vacuum infiltrated for 30 min, and then incubated at 37°C for 4 h. Stained tissues were destained in successive baths of 50, 60, 80, 95, and 100% ethanol and then observed with a Zeiss stereomicroscope.

### Trichome Isolation and Protein Extraction

Seeds were first sown on agar plates for 2 weeks and then transferred to hydroponic cultivation (one-quarter-strength Hoagland nutrient solution). After 1 week, the plants were exposed to Fe-deficient conditions for 1 week. Trichomes were isolated as described previously (Wang et al., 2001; Van Cutsem et al., 2011). Briefly, young leaves were removed and immediately cooled on ice packs precooled at −80°C. The leaves were then dipped in liquid nitrogen and carefully scratched with a spatula to remove the trichomes, which were collected in a liquid nitrogen pool. The isolated trichomes were filtered through a 350-µm nylon mesh.

The isolated trichomes were resuspended with 1 ml deionized water and then 50 µl of the suspension was used to extract the total proteins. The rest of the trichomes were used to measure the metal content. Total protein of the trichomes were extracted as previously described (Van Cutsem et al., 2011).

### Elemental Analysis

The shoots and roots of plants were harvested separately. Shoots were washed twice with deionized water. Roots were desorbed by washing them for 10 min with 2 mM CaSO_4_ and 10 mM EDTA and then washed twice in deionized water. Samples were dried at 70°C for 3 days and then digested in HNO_3_ at 120°C for 45 min using a microwave digester (ETHOS One, Milestone). Metal content was measured by ICP-MS (Optima 8000, PerkinElmer).

### Chlorophyll Measurements

Chlorophyll concentrations were determined following extraction in N,N-dimethylformamide (Eroglu et al., 2016).

### Yeast Transformation and Growth Analyses

The following strains of *Saccharomyces cerevisiae* were used: wild-type parental strain BY4741, ferrous Fe uptake-defective double mutant *Δfet3fet4*, and Fe-sensitive mutant *Δccc1*. Yeast transformation was performed using the lithium acetate/PEG transformation method (Gietz et al., 1995). Positive colonies were selected on synthetic dropout (SD) plates containing a synthetic defined medium without uracil (pH 6). Yeast strains carrying an empty vector (pYES2) or expressing AtNRAMP6 were pre-cultured in SD-Ura liquid medium at 30°C for 16 h. Pre-cultured cells were centrifuged and diluted into sterile water to an OD_600_ of 1.0, and 10μl aliquots were spotted onto SD-Ura plates containing 50 μM bathophenanthroline disulfonic acid (BPDS) or various metals at different concentrations. The plates were incubated at 30°C for 3 d.

### Bioinformatics Analysis of AtNRAMP6

AtNRAMP6 (GenBank Acc. No. At1g15960) and its homologues were aligned using Multalin (Corpet, 1988). The 3D model of AtNRAMP6 was generated by homology modeling using SWISS-MODEL (http://swissmodel.expasy.org/) based on the structure of ecoDMT (PDB ID 5m87.1.A). Images were generated by using PyMOL 1.6.x.

## Results

### Structure of AtNRAMP6

Consistent with results from a previous study (Cailliatte et al., 2009), two AtNRAMP6 transcript isoforms were cloned from the total cDNA, which represents the full-length transcript (corresponding to the sequence of At1g15960 from TAIR) and a transcript isoform that contains unspliced intron 6. The isoform introduces a stop codon 36 nucleotides downstream of the exon6–intron6 junction. In this way, the long *AtNRAMP6* transcript encodes the full-length AtNRAMP6 protein (amino acids 1–527), and the short *AtNRAMP6* transcript encodes a truncated AtNRAMP6 protein (amino acids 1–180). It has been demonstrated that only the full-length AtNRAMP6 protein mediates cadmium toxicity in yeast, but the truncated AtNRAMP6 protein was shown to be nonfunctional (Cailliatte et al., 2009). This study further investigates the function of the full-length AtNRAMP6 protein.

AtNRAMP6 was aligned with other NRAMP proteins, including EcoDMT, AtNRAMP1, AtNRAMP2, AtNRAMP3, AtNRAMP4, OsNRAMP1, and OsNRAMP5. AtNRAMP6 shares up to 81.5% identity with AtNRAMP1. Three-dimensional modeling of AtNRAMP6 showed a strong bias towards *Eremococcus coleocola* DMT (5m87.1.A) through the use of the SWISS-MODEL server (http://swissmodel.expasy.org), which indicated that AtNRAMP6 contains 12 transmembrane helices and a conserved metal binding site (D54, N57, A226, and M229 in AtNRAMP6) (**Figure S1**).

### Expression Pattern of AtNRAMP6

**Figure 1:**
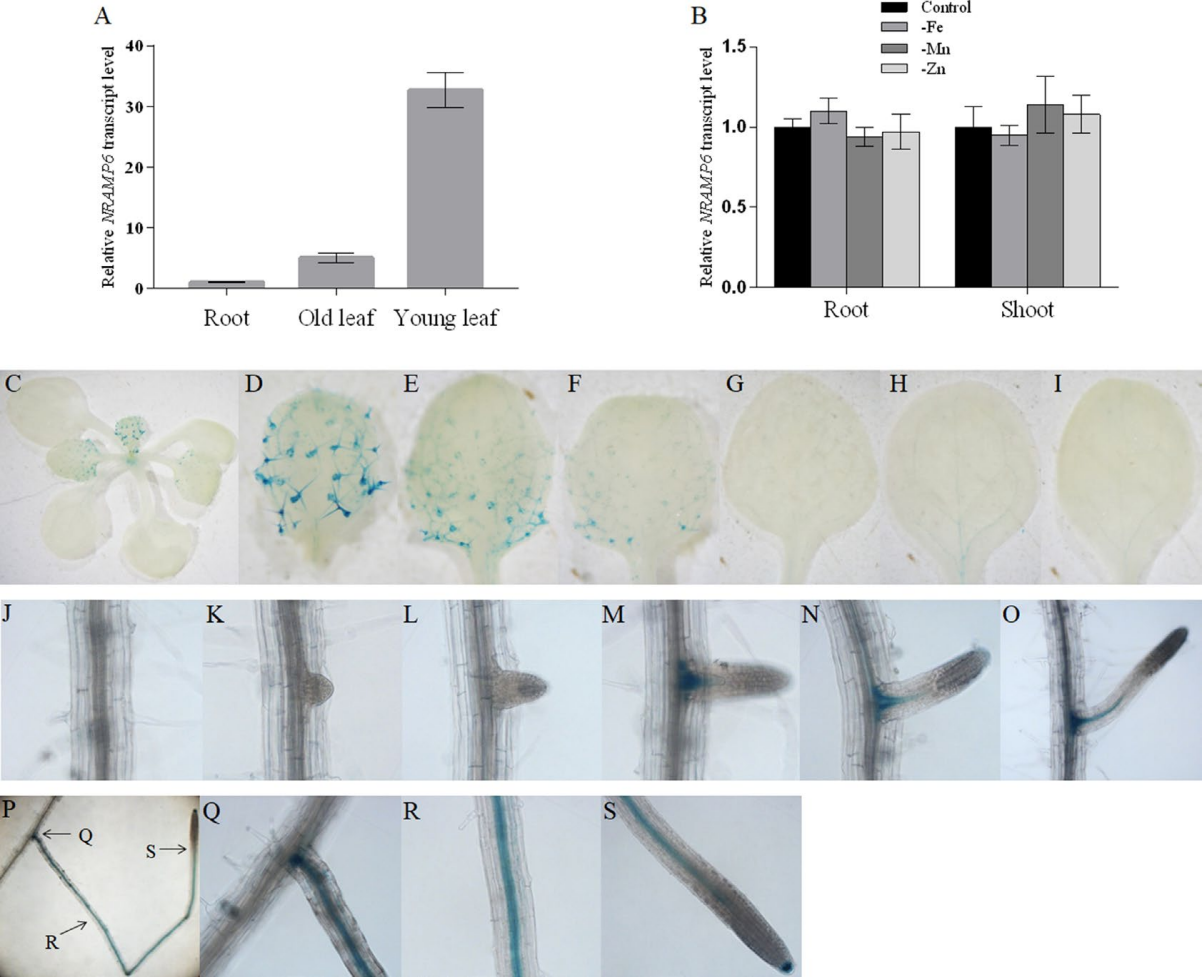
Expression pattern of AtNRAMP6. **(A)** The expression levels of *AtNRAMP6* in different tissues of 4-week-old wild-type plants. Data represents the mean ± SD (n = 3). **(B)** Expression analysis of *AtNRAMP6* in roots and shoots in response to Fe, Mn, or Zn deficiency. Wild-type plants were sown on agar plates for 2 weeks, transferred to hydroponic cultivation for 1 week followed by 7 d in control or in Fe-, Mn-, or Zn-deficient conditions. Data represents the mean ± SD (n = 3). (C-S) Histochemical staining of GUS activity in proNRAMP6:GUS-transformed Arabidopsis. Plants were grown in Fe-replete conditions for 7 d and then harvested for staining. **(C)** Shoot. **(D**–**F)** Young leaves. **(G**–**I)** Old leaves. **(J)** Primary root. **(K**–**S)** Lateral root.

Quantitative real-time PCR (qRT-PCR) was used to determine whether *AtNRAMP6* was expressed in various organs of *Arabidopsis* grown hydroponically. We found that *AtNRAMP6* transcripts in the shoot tissues were much higher than that in the roots of plants grown for 3 weeks in hydroponic culture, and the transcripts in the young leaves (three to four top leaves) were higher than those in the old leaves (the remainder of leaves below the young leaves) (**Figure 1A**). In addition, Mn, Fe, or Zn deficiency did not significantly affect *AtNRAMP6* expression in either the roots or the shoots (**Figure 1B**).

In order to confirm the tissue-specific expression of *AtNRAMP6* within different plant organs, transgenic *Arabidopsis* lines were transformed with the Pro*NRAMP6:GUS* (β-glucuronidase) construct. One representative line out of 12 primary transformants was selected to analyze the GUS activity. GUS staining of seedlings showed that young leaves exhibited higher GUS expression than old leaves did, and strong GUS activity was observed in the trichomes of young leaves (**Figures 1C–I**). In the roots, staining was extremely weak in the main roots, whereas strong GUS expression was observed in the lateral roots (**Figures 1J–S**). Interestingly, no staining was revealed in the immature lateral roots during either the lateral root primordia (LRP) initiation stage or the emergence stage (**Figures 1J–L**). The staining appeared with the development of the lateral root and was restricted to the stele and root cap (**Figures 1M–S**).

### Phenotypic Analysis of the AtNRAMP6 Mutation

**Figure 2:**
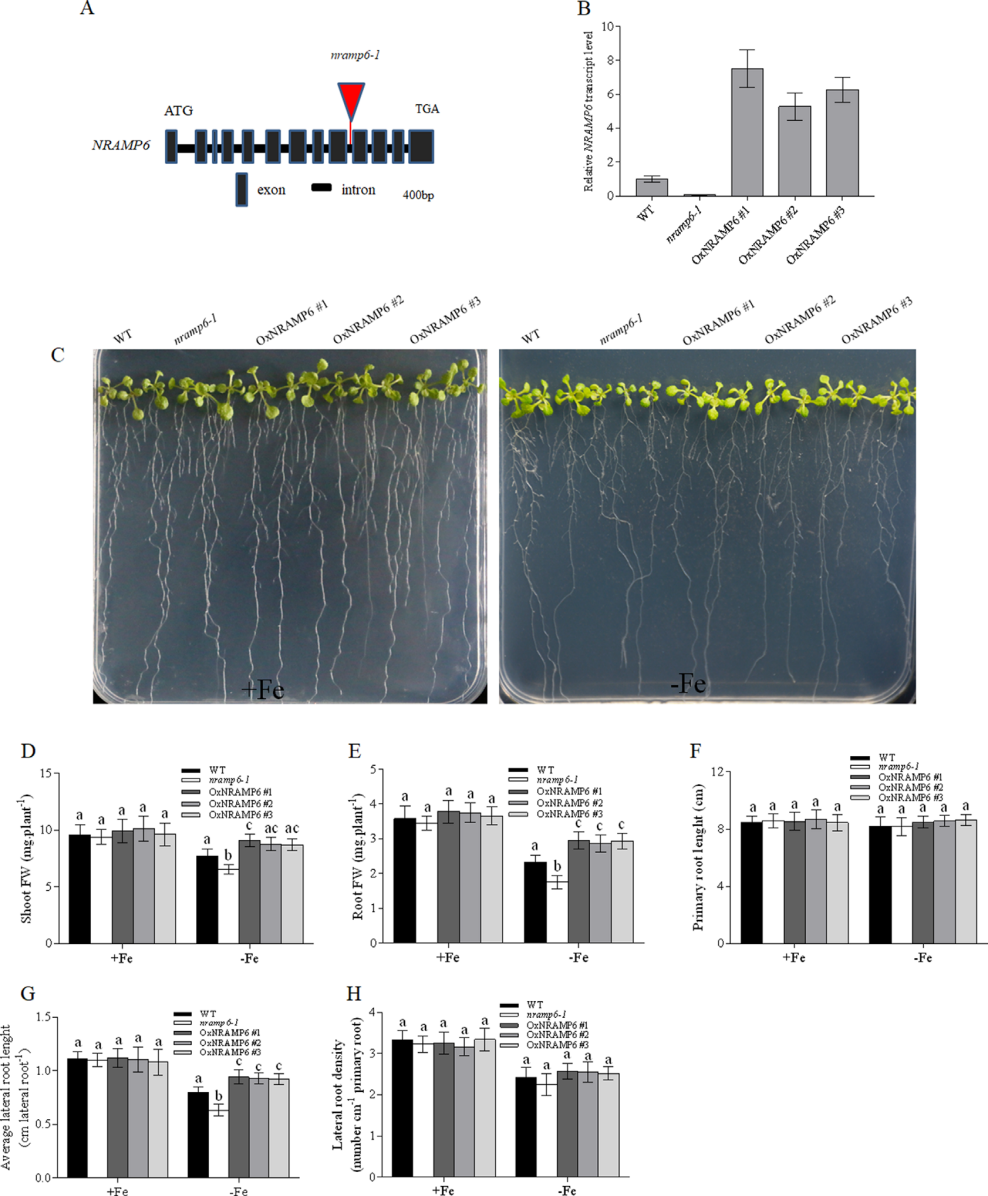
Knockout of *NRAMP6* impaired lateral root growth under Fe-deficient conditions. **(A)** The T-DNA was inserted in the 9th intron of the *NRAMP6* gene. **(B)** Relative *NRAMP6* transcript levels in wild-type, *nramp6-1*, and three complemented lines OxNRAMP6 #1, OxNRAMP6 #2, and OxNRAMP6 #3, assessed by quantitative RT-PCR. *Actin2* was used as an internal control. Data represent the mean ± SD (n = 3). **(C)** Phenotypic analysis of the indicated plants in response to Fe-deficient conditions. WT, *nramp6-1*, and complemented lines were sown on a one-half-strength Hoagland agar medium either in Fe-replete conditions (20µM Fe[III]-EDTA) or in Fe-free (no added Fe) conditions as indicated. Plants were grown vertically for 14 days. (D-H) Shoot fresh weight **(D)**, root fresh weight **(E)**, primary root length **(F)**, average lateral root length **(G)**, lateral root density **(H)**. Data are means ± SD. Means with different letters are significantly different (P < 0.05, Tukey’s test).

To investigate the physiological function of *AtNRAMP6* in the plant, we obtained a T-DNA insertion mutant named *nramp6-1* from TAIR and inserted it in the 9^th^ intron (**Figure 2A**). The *AtNRAMP6* transcript was not detected in this line (**Figure 2B**), indicating that this is a knockout line of *AtNRAMP6*. In addition, we generated three complemented *nramp6* lines in the *nramp6-1* mutant background, named OxNRAMP6 #1, OxNRAMP6 #2, and OxNRAMP6 #3, respectively (**Figure 2B**).

We found no apparent phenotype of the *nramp6-1* mutant when the plant was grown in nutritionally adequate conditions. To investigate the role of *AtNRAMP6* on plant growth, we grew WT, *nramp6-1*, and complemented lines on agar plates in Fe-deficient conditions for 2 weeks. The growth of *nramp6-1* was more inhibited compared with the WT (**Figure 2C**). The biomass of the shoots and roots in the knockout line was 70% and 77% of the wild type, respectively, of the wild type (**Figures 2D, E**). The primary root length was not different between *nramp6-1* and WT (**Figure 2F**); however, the lateral root length of *nramp6-1* was significantly reduced compared with that of the WT (**Figure 2G**), and there was no change in the lateral root number (**Figure 2H**). The phenotype was fully reverted in the complemented lines compared with the knockout line (**Figures 2C–H**), confirming that the mutation in AtNRAMP6 gene is responsible for the phenotype observed in the mutant. The growth of *nramp6-1* did not differ from that of the WT under toxic concentrations of Fe (**Figure S2**). These results indicated that AtNRAMP6 plays an important role in plant growth under Fe-deficient conditions.

**Figure 3:**
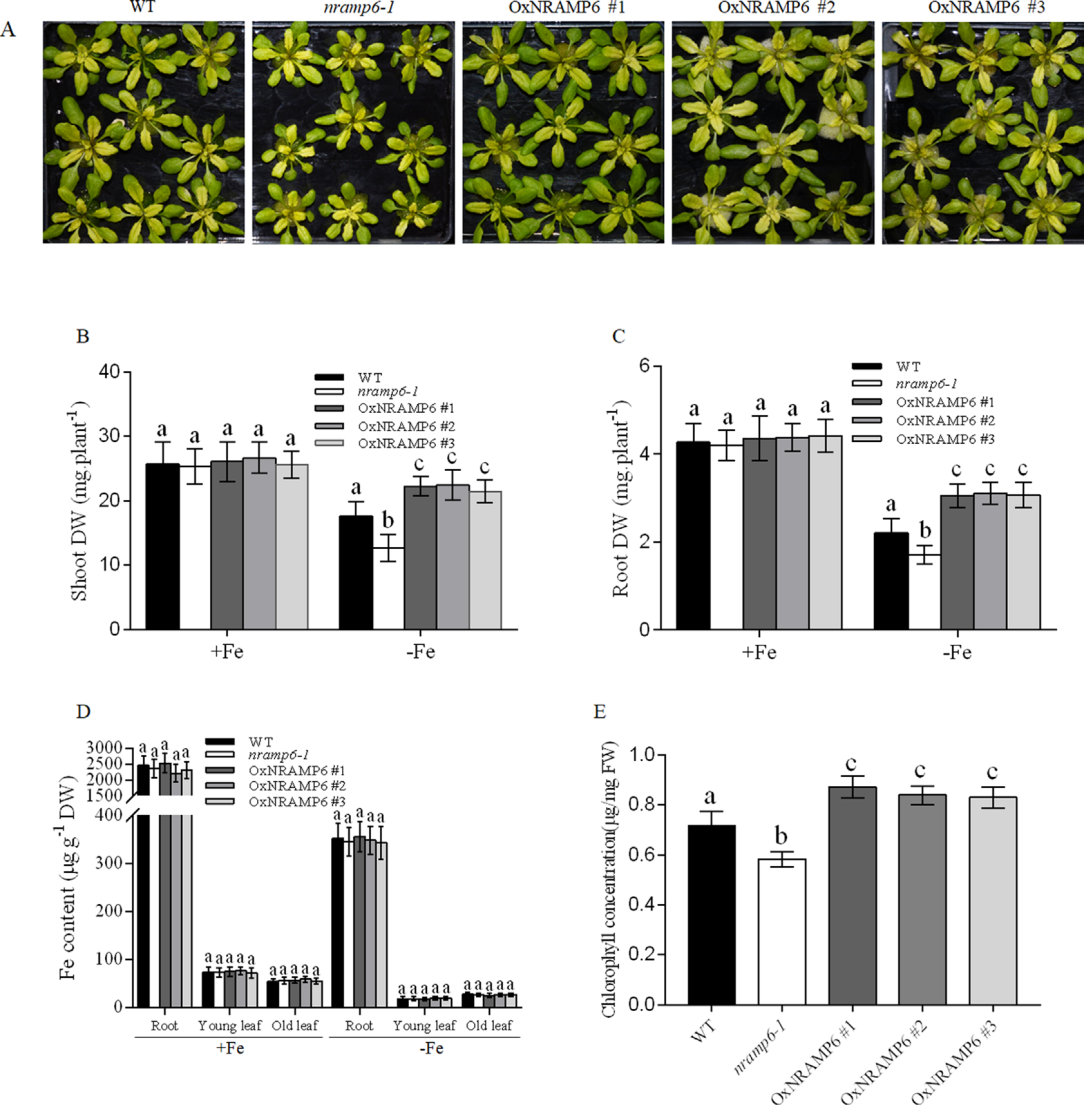
Effect of the *NRAMP6* mutation on Fe accumulation and chlorophyll content in Arabidopsis thaliana. **(A)** Phenotypes of plants grown in hydroponic conditions. WT, *nramp6-1*, and complemented lines were sown on agar plates for 2 weeks and then transferred to hydroponic cultivation for 1 week in Fe-replete conditions followed by 7 d in Fe-replete or Fe-deficient conditions. **(B**-**E)** Dry weight of roots **(B)** and shoots **(C)**. Fe contents in roots and young leaves (three to four top leaves), and old leaves (the rest of leaves) **(D)**. Chlorophyll levels of WT, *nramp6-1*, and three overexpression lines **(E)**. Data represent the mean ± SD of three biological replicates. Means with different letters indicate a significant difference at P < 0.05 using Tukey’s test.

To examine whether the metal content in plants was influenced by the *nramp6* mutation, the wild-type, *nramp6-1*, and complemented lines were sown on agar plates for 2 weeks and then transferred to hydroponic cultivation. After 1 week, the plants were exposed to Fe-deficient conditions for 1 week. Results showed that the growth of the *nramp6-1* was inhibited in Fe-deficient conditions (**Figure 3A**), and the dry weight of the roots and shoots were reduced in the *nramp6-1* (**Figures 3B, C**). The complemented lines show full reversion of the *nramp6-1* phenotype in Fe-deficient growth conditions (**Figures 3A–C**). The metal concentrations in roots and leaves were measured using inductively coupled plasma-mass spectrometry (ICP-MS). Results showed that Fe concentration in the roots, young leaves, and old leaves did not differ between these lines in either Fe-replete or Fe-deficient conditions (**Figure 3D**). In addition, the amount of Mn, Zn, and Cu was also not modified in the mutant (**Figure S3**). Furthermore, we measured the Fe concentrations in secondary roots and in the trichomes of young leaves. Results showed that the Fe concentration did not significantly decrease or increase in *nramp6-1* compared with WT plants, either in secondary roots or in isolated trichomes (**Figure S4**).

We next measured the Chlorophyll content in the wild-type, *nramp6-1* and complemented lines which grown in Fe-deficient conditions. The results showed that the chlorophyll concentration was decreased in *nramp6-1*, while the concentration in complemented lines was greater than that in WT (**Figure 3E**). These data indicate that AtNRAMP6 is conducive to the synthesis of chlorophyll.

### Subcellular Localization of AtNRAMP6

**Figure 4:**
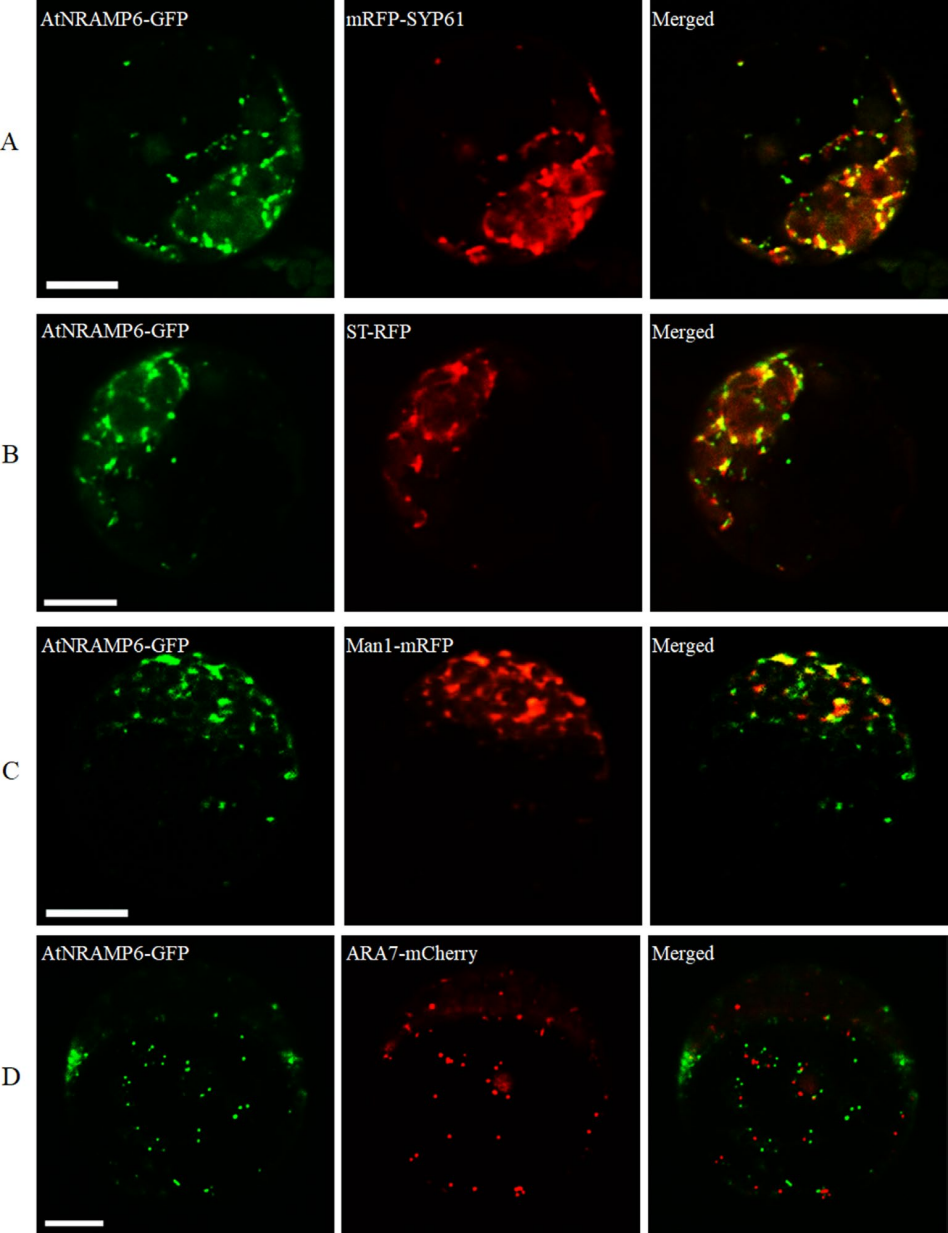
Subcellular localization of AtNRAMP6-GFP fusion protein in Arabidopsis protoplast. The AtNRAMP6-GFP construct was co-transformed with the marker mRFP-SYP61 (TGN) **(A)**, ST-RFP (Golgi) **(B)**, Man1-mRFP (cis-Golgi) **(C)**, and mRFP-ARA7 (PVC) **(D)**. Scale bars: 10 µm.

To determine the subcellular localization of AtNRAMP6, we transiently expressed a *AtNRAMP6-GFP* fusion gene in protoplasts prepared from *Arabidopsis* leaves. Fluorescence in the cells expressing the fusion gene was observed inside the cell as numerous small motile structures. To identify the compartment to which AtNRAMP6 was localized, *AtNRAMP6-GFP* was co-expressed with markers for different internal compartments in *Arabidopsis* mesophyll protoplasts. The AtNRAMP6–GFP fluorescence signals overlapped significantly with the TGN marker mRFP-SYP61 (**Figure 4A**), indicating high colocalization between the two proteins. Colocalization between AtNRAMP6 and two Golgi marker (trans-Golgi cisternae marker ST-RFP and cis-Golgi marker Man1-mRFP) were also substantial (**Figures 4B, C**), suggesting that AtNRAMP6 may also reside in the Golgi. However, no colocalization was seen between AtNRAMP6-GFP and mRFP-ARA7 (a prevacuolar compartment marker) (**Figure 4D**). Moreover, upon heterologous expression in *S. cerevisiae*, AtNRAMP6::HA was located in an endomembrane compartment that is different from that of a vacuole or mitochondrion (Cailliatte et al., 2009). These results indicate that AtNRAMP6 is associated with the TGN and Golgi.

### AtNRAMP6 Has Fe Transport Activity in Yeast

**Figure 5:**
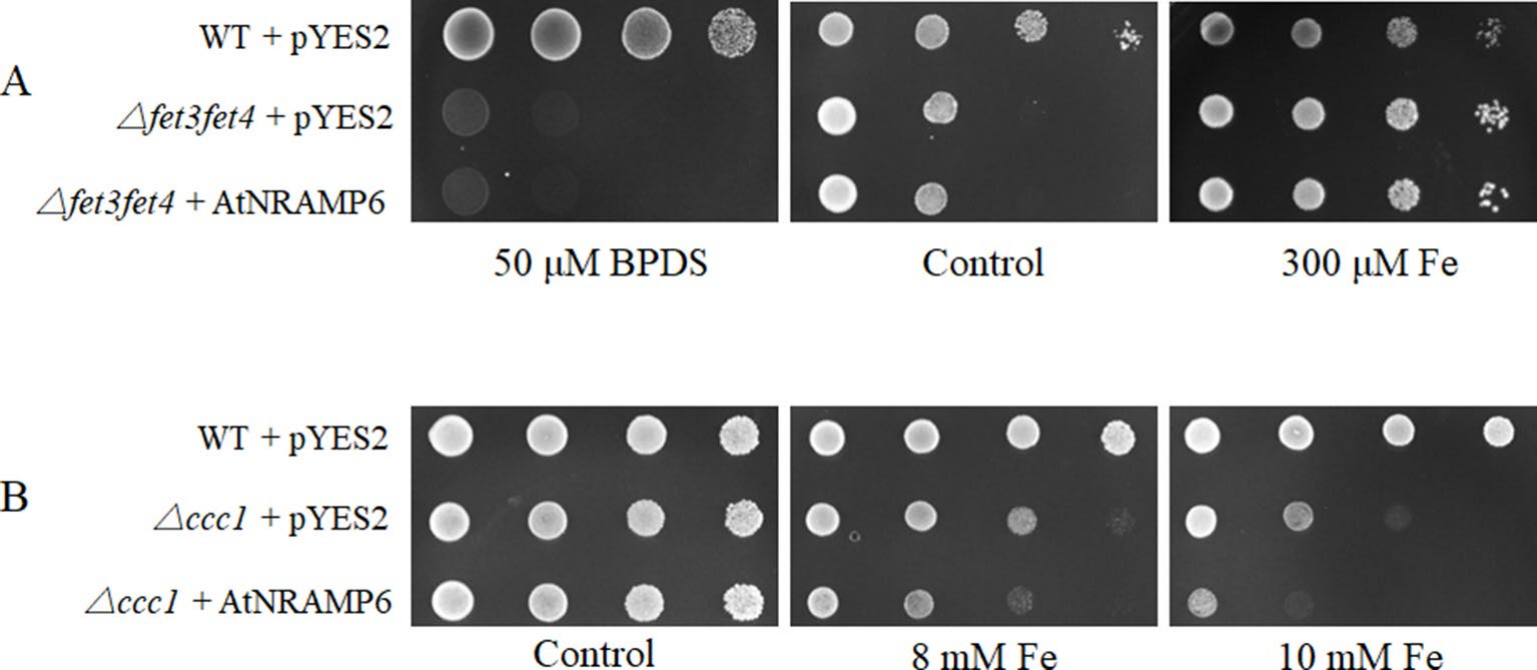
Expression of AtNRAMP6 increased the sensitivity of *Δccc1*. Wild-type and mutant yeast strains containing the empty vector or AtNRAMP6 were spotted onto synthetic dropout (SD)-Ura plates with BPDS or metal supplementation as indicated. **(A)** AtNRAMP6 fails to complement the phenotype of the yeast mutant *Δfet3fet4* without supplemented with Fe. **(B)** The expression of AtNRAMP6 exacerbated the sensitivity of the yeast mutant *Δ*ccc1 to excessive Fe.

To examine the metal transport activity of AtNRAMP6, we tested whether the AtNRAMP6 is able to complement a yeast Fe-transport mutant, *Δfet3fet4*, which is defective in both low- and high-affinity Fe uptake systems (Dix et al., 1994). Results showed that AtNRAMP6 was unable to complement the phenotype of Fe uptake in the yeast mutant *Δfet3fet4* (**Figure 5A**), a result that is consistent with previous research (Cailliatte et al., 2009). When we conducted metal toxicity growth assays using the yeast mutant ▵*ccc1*, which is sensitive to excessive Fe. The results showed that the expression of *AtNRAMP6* increased the sensitivity of the ▵*ccc1* to high Fe (**Figure 5B**), which indicates that AtNRAMP6 participates in Fe-transport activity in yeast. In addition, we test the function of the AtNRAMP6-GFP fusion protein in yeast. The results showed that the AtNRAMP6-GFP fusion protein is functional when expressing it in the *ccc1* yeast mutant (**Figure S5**).

## Discussion

Fe deficiency is known to reduce chlorophyll synthesis and photosynthetic activity and cause chlorosis and decreased biomass in plants (Graziano et al., 2002). In *Arabidopsis*, IRT1 is a high-affinity Fe transporter that promotes Fe uptake under Fe-deficient conditions (Vert et al., 2002; Barberon et al., 2011). AtNRAMP3 and AtNRAMP4 release Fe from vacuoles to sustain plant growth under Fe-limited conditions (Thomine et al., 2003; Lanquar et al., 2005; Mary et al., 2015). However, the intracellular Fe distribution that facilitates plant growth in Fe-deficient conditions is seldom studied. In this study, we show that AtNRAMP6 localized to the Golgi and TGN. We also found evidence showing that AtNRAMP6 is required for lateral root growth in low Fe conditions.

### AtNRAMP6 Expression in Lateral Roots

Plant root systems perform many essential adaptive functions, such as water and nutrient uptake. The lateral root is an important part of a plant root system (Sanchez-Calderon et al., 2005). Root development is highly sensitive to environmental cues, and the supply of nutrients such as nitrogen (N), phosphorus (P), and Fe have a major influence on root growth and architecture (Zhang and Forde, 1998; Zhang et al., 1999; Lopez-Bucio et al., 2003). Symplastic Fe triggered the local elongation of lateral roots (Giehl et al., 2012).

The results of qRT-PCR show that *AtNRAMP6* transcripts in the shoot tissues was much higher than that in the roots, and young leaves exhibited higher *AtNRAMP6* expression than old leaves did. These results were consistent with that in a previous research (Cailliatte et al., 2009). Although the GUS staining in the main root was extremely weak, but strong GUS expression was nonetheless observed in the stele of the lateral roots. In addition, GUS activity was neither found in the LRP nor in the immature lateral root. This expression pattern might explain why lateral root length was reduced even though there was no change in the lateral root number in the *nramp6-1* mutant and no difference in the main root length between *nramp6-1* and WT under Fe-deficient conditions.

### AtNRAMP6 Localized to the Golgi and TGN

We found that AtNRAMP6 was unable to complement the phenotype of Fe uptake in the yeast mutant *Δfet3fet4*. When we conducted a metal toxicity growth assay using the yeast mutant *Δccc1*, which is sensitive to Fe, the expression of *AtNRAMP6* exacerbated the sensitivity to excessive Fe. AtNRAMP2 was recently reported to be a manganese transporter localized to the TGN, but it nonetheless failed to recover the plasma membrane Mn uptake of the yeast *Δsmf1* mutant (Alejandro et al., 2017; Gao et al., 2018). We therefore propose that AtNRAMP6 works intracellularly in the plant. When transiently expressed as an *AtNRAMP6-GFP* fusion gene in *Arabidopsis* protoplasts, the fluorescence appeared inside the cell as numerous small motile structures. Using a set of endomembrane markers, we showed that AtNRAMP6 colocalized with the TGN marker SYP61 and the Golgi markers ST-RFP and Man1-mRFP. Some ion transporters associated with the Golgi and endosomal compartments have been discovered, including the Golgi- and TGN-localized inorganic phosphate transporter PHO1 and the Na^+^/H^+^ antiporters AtNHX5 and AtNHX6 which both localized to the Golgi and TGN (Bassil et al., 2011; Arpat et al., 2012). In plants, TGN plays an important role in post-Golgi trafficking, as it merges the exocytic and endocytic pathways (Uemura and Nakano, 2013). OsMTP11 was identified as a TGN-localized Mn transporter that is crucial for Mn homeostasis and tolerance in rice (Ma et al., 2018). AtIRT1 and AtNRAMP1 were found to perform dual targeting between the plasma membrane and the intracellular vesicles of the endomembrane pathway (Barberon et al., 2011; Agorio et al., 2017), while AtNRAMP2 was found to be a resident TGN protein (Alejandro et al., 2017; Gao et al., 2018).

### AtNRAMP6 Plays an Important Role in Fe Homeostasis

NRAMPs have been shown to transport a wide range of divalent metal ions (Nevo and Nelson, 2006; Socha and Guerinot, 2014). AtNRAMP1, AtNRAMP3, and AtNRAMP4 were reported to be able to transport Mn, Fe, and Cd (Thomine et al., 2003; Lanquar et al., 2005; Lanquar et al., 2010; Castaings et al., 2016). AtNRAMP2 has a role in the intracellular distribution of Mn and in the activity of Fe and Zn in yeast (Alejandro et al., 2017; Gao et al., 2018). AtNRAMP6 has been described as having the ability to transport Cd, but its physiological function is currently unclear (Cailliatte et al., 2009).

The Golgi/TGN localization of AtNRAMP6 indicates that AtNRAMP6 is unlikely to perform Fe uptake through the plasma membrane, which might explain why AtNRAMP6 cannot complement the plasma membrane Fe uptake defect of the yeast mutant *Δfet3fet4*. Interestingly, we found that AtNRAMP6 exacerbated the sensitivity of *Δccc1* to excessive Fe, suggesting that AtNRAMP6 can transport Fe. *Ccc1* is localized to the vacuole and involved in the transfer of Fe from the cytosol to the vacuole, while *Δccc1* is hypersensitive to high Fe due to increased Fe accumulation in the cytosol (Li et al., 2001). Based on these results, we propose that AtNRAMP6 might mediate Fe import into the cytosol.

Among *Arabidopsis* NRAMP family members, AtNRAMP1, AtNRAMP3, and AtNRAMP4 have been shown to be involved in Fe transport in plants. However, unlike AtNRAMP6, these proteins perform Fe-transport functions using different mechanisms. AtNRAMP1 plays a pivotal role in Fe transport by cooperating with IRT1 to take up Fe in roots under Fe-replete conditions (Castaings et al., 2016). Conversely, AtNRAMP3 and AtNRAMP4 are localized to the tonoplast participate, specifically in Fe mobilization from vacuolar metal stores during seed germination (Thomine et al., 2003; Lanquar et al., 2005), yet the expression of *AtNRAMP6* has been shown to exacerbate the sensitivity of the yeast mutant Δccc1 to excessive Fe and to combine with the phenotypes of the *nramp6-1* mutant and complemented lines. Therefore, we propose that AtNRAMP6 is involved in the transport of Fe from the Golgi/TGN vesicles to the cytosol for Fe homeostasis under Fe-deficient conditions.

Plant root development depends on nutrient availability; Fe plays an important role in regulating root elongation. The research indicated that spatially restricted Fe availability in root tissues had a particularly strong impact on the elongation of lateral roots and that the impact of Fe on lateral root development is primarily subject to a root-endogenous systemic regulation (Giehl et al., 2012). A mitochondrial protein, OsSPR1, plays an important role in Fe homeostasis and is involved in lateral root elongation (Jia et al., 2011). We found that AtNRAMP6 is mainly expressed in lateral roots and located in the intracellular membrane system; mutation of *AtNRAMP6* caused impaired lateral root growth under Fe deficiency. These observations suggest that AtNRAMP6 may be related to the Fe releases from Golgi/TGN to cytosol and hence contributes to the Fe reutilization. A mutation in *AtNRAMP6* likely to disturb Fe homeostasis and thus inhibits lateral root growth.

In conclusion, our results show that AtNRAMP6 is a Golgi- and TGN-localized transporter involved in Fe homeostasis and required for the lateral roots growth in *Arabidopsis*. And the results suggest that the Golgi may be an essential component for the storage of Fe in *Arabidopsis*.

## Data Availability

All datasets generated for this study are included in the manuscript/supplementary files.

## Author Contributions

JiyL, XC, and WZ conceived and designed the experiments. JiyL, YW, LZ, YLi, and JinL conducted experiments. XZ, DG, EX, and YLu contributed new reagents or analytical tools. JiyL, YW, and XC analyzed data. JiyL, XC, and WZ wrote the manuscript. All authors read and approved the manuscript.

## Funding

This work was supported by the National Natural Science Foundation of China [grant numbers 31301839, 51572131], the Natural Science Foundation of Jiangsu Province of China [grant number BK20130672], and a project funded by the Fundamental Research Funds for the Central Universities [grant number KJZ201743].

## Conflict of Interest Statement

The authors declare that the research was conducted in the absence of any commercial or financial relationships that could be construed as a potential conflict of interest.
