# Supplementary material for: The Intracellular Transporter AtNRAMP6 Is Involved in Fe Homeostasis in Arabidopsis
Source: Front Plant Sci. 2019 Sep 17;10:1124. doi: 10.3389/fpls.2019.01124 (PMC6757856; doi:10.3389/fpls.2019.01124)
Supplement: Supplementary file 1 [file DataSheet_1.pdf]

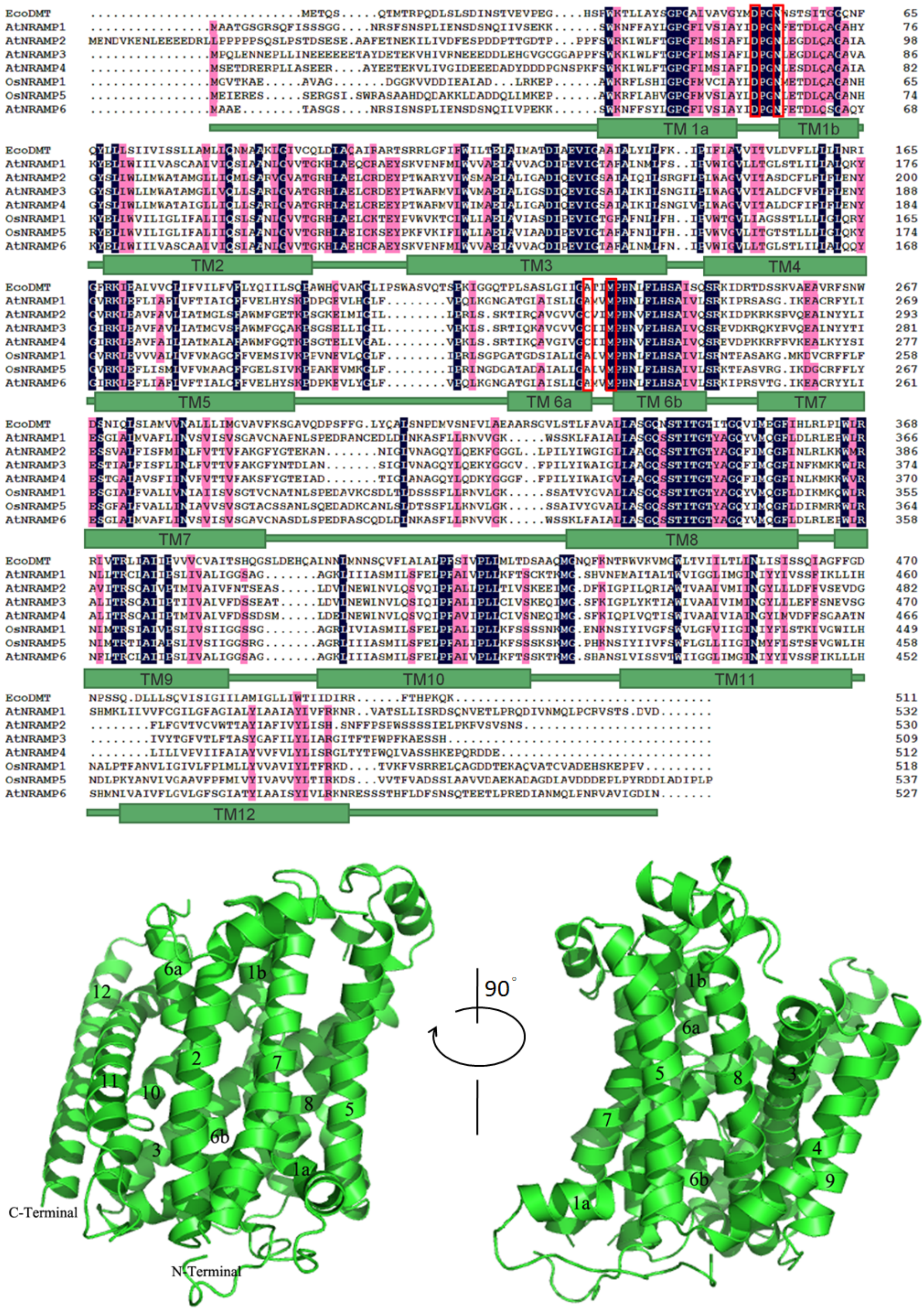

Figure S1. Structure of AtNRAMP6.

(A) Amino acid sequence alignment of AtNRAMP6 with other NRAMPs. AtNRAMP1 (GenBank Acc. No. At1g80830), AtNRAMP2 (GenBank Acc. No. AT1G47240), AtNRAMP3 (GenBank Acc. No. AT2G23150), AtNRAMP4 (GenBank Acc. No. AT5G67330), and AtNRAMP6 (GenBank Acc. No. At1g15960) from Arabidopsis thaliana and OsNRAMP1 (Phytozome: LOC_ Os07g15460) and

OsNRAMP5 (Phytozome: LOC_ Os07g15370) from Oryza sativa and Eremococcus coleocola EcoDMT (UniProtKB identifier E4KPW4) were aligned. Identical residues are highlighted in mazarine, while similar residues are highlighted in pink. The putative transmembrane regions of the AtNRAMP6 were predicted based on the alignment with EcoDMT and are shown below the sequences. Selected residues of ion-binding site are indicated within a red frame. (B) 3D model of AtNRAMP6. A 3D model of the AtNRAMP6 was rendered using SWISSMODEL based on the X-ray crystal structure of EcoDMT (PDB ID 5m87.1.A). The model is shown in two different aspects.

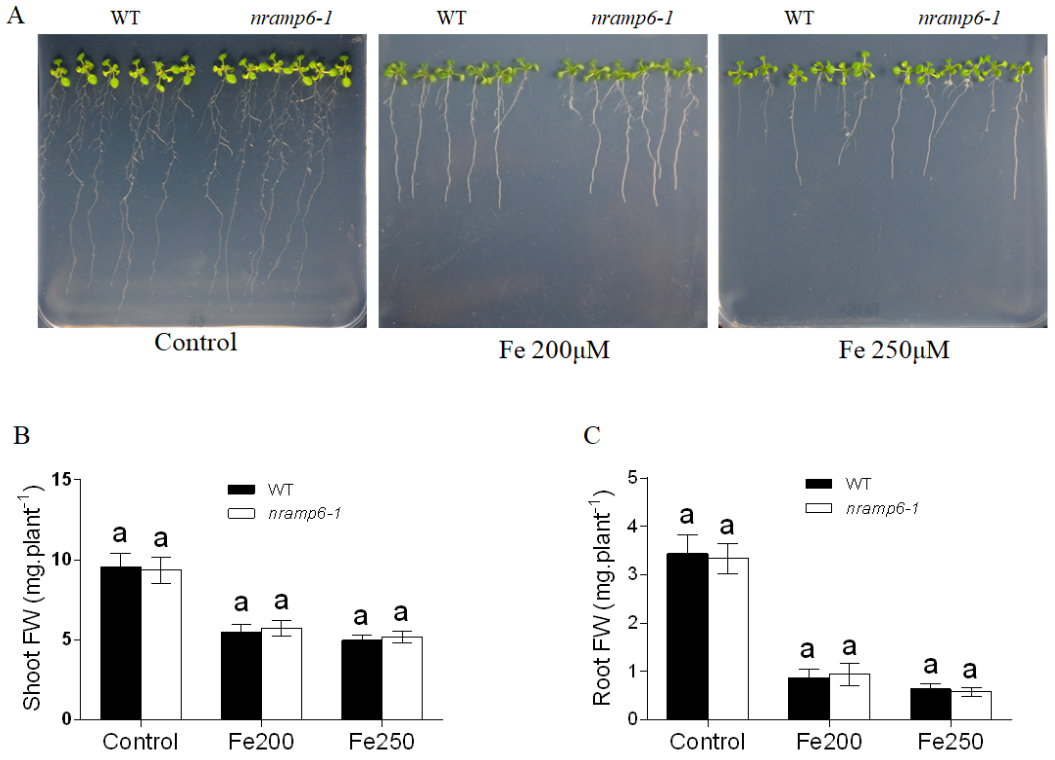

Figure S2. Response of *nramp6-1* to Fe toxicity.

(A) WT and *nramp6-1* were sown on a one-half-strength Hoagland agar medium either in control conditions or in Fe supplementation conditions as indicated. Plants were grown vertically for 14 d. (B) Shoot fresh weight **(C)** root fresh weight.

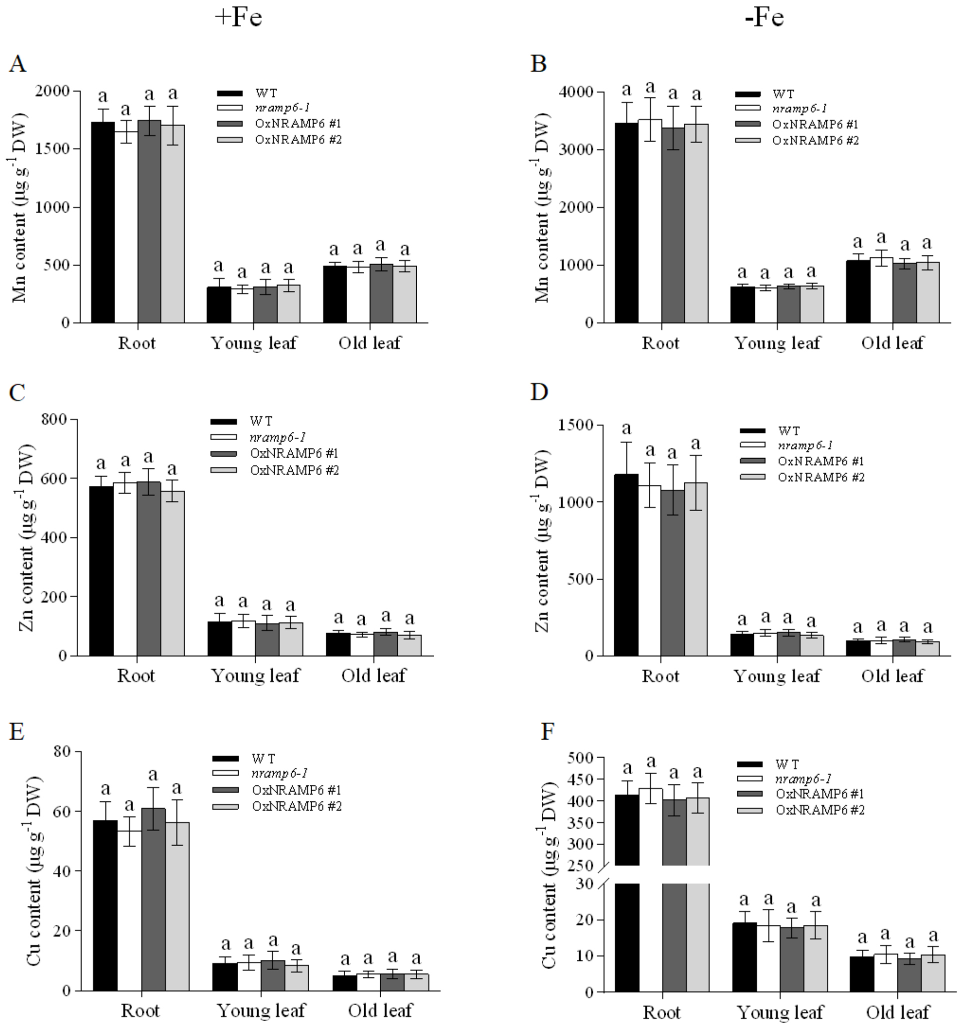

Figure S3. Effect of the *nramp6* mutation on Mn, Zn, or Cu accumulation in *Arabidopsis thaliana*.

WT and *nramp6-1* were sown on agar plates for two weeks and then transferred to hydroponic cultivation for one week in Fe-replete conditions followed by 7 days in Fe-replete (A, C, E) or Fe-deficient (B, D, F) conditions. Roots, young leaves, and old leaves were harvested. Mn (A, B), Zn (C, D) and Cu (E, F) contents were determined. Data represent the mean ± SD of three biological replicates. Means with different letters indicate a significant difference at $P < 0.05$ using Tukey's test.

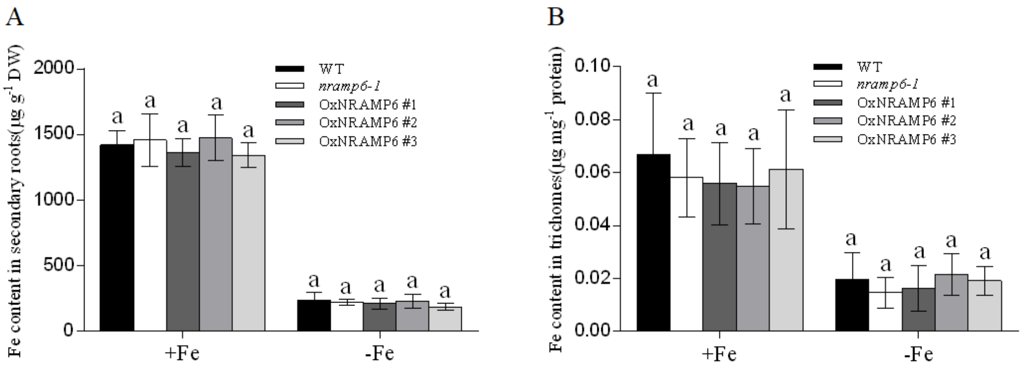

Figure S4. Effect of the *nramp6* mutation on Fe accumulation in secondary roots and in the trichomes of young leaves.

WT, *nramp6-1* and complemented lines were sown on agar plates for two weeks and then transferred to hydroponic cultivation for one week in Fe-replete conditions followed by 7 d in Fe-replete or Fe-deficient conditions. The secondary roots and the trichomes of young leaves were collected to measure the Fe concentration. (A) Fe concentration in secondary roots, (B) Fe concentration in trichomes. Data represent the mean ± SD of three biological replicates. Means with different letters indicate a significant difference at $P < 0.05$ using Tukey's test.

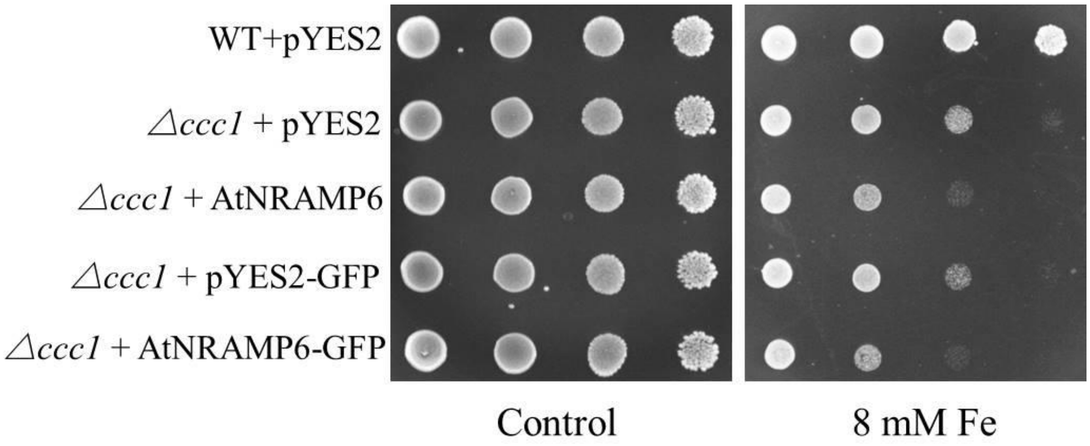

Figure S5. AtNRAMP6-GFP constructs showed similar transport activity with non-GFP tagged proteins.

Wild-type and mutant yeast strains containing the empty vector, AtNRAMP6 or AtNRAMP6-GFP were spotted onto synthetic defined (SD)-Ura plates with metal supplementation as indicated. Transformed cells were grown in liquid SD medium supplemented with 8 mM Fe^{2+} at an initial OD600 = 0.1.
